# Supplementary material for: Aspartic Acid Isomerization Characterized by High Definition Mass Spectrometry Significantly Alters the Bioactivity of a Novel Toxin from Poecilotheria
Source: Toxins (Basel). 2020 Mar 25;12(4):207. doi: 10.3390/toxins12040207 (PMC7232244; doi:10.3390/toxins12040207)
Supplement: Supplementary file 1 [file toxins-12-00207-s001.pdf]

Supplementary Materials: Aspartic Acid Isomerization Characterized by High Definition Mass Spectrometry Significantly Alters the Bioactivity of a Novel Toxin from *Poecilotheria*

Stephen R. Johnson * and Hillary G. Rikli

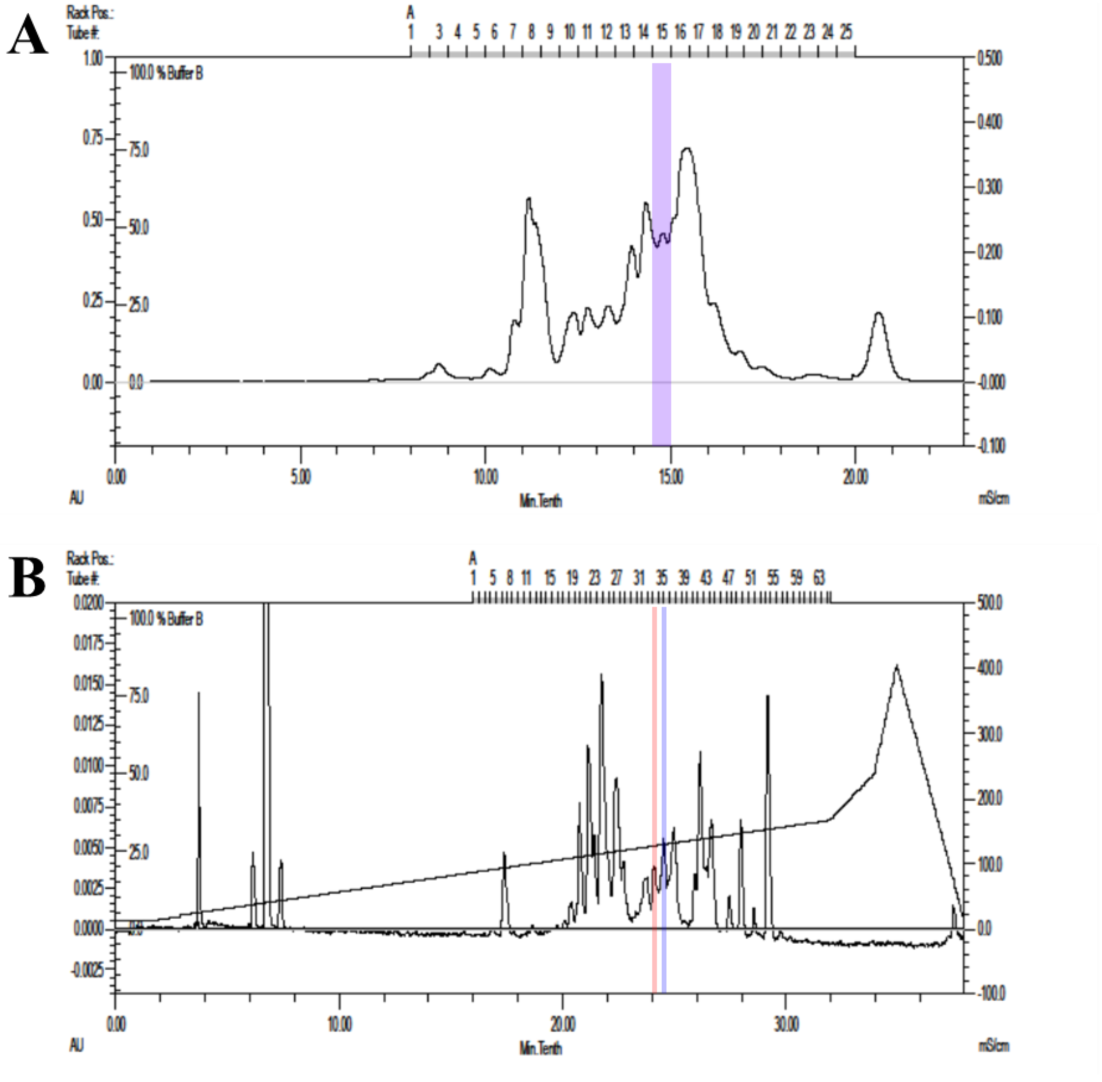

Figure S1. Representative purification chromatograms. (**A**) Size exclusion chromatography was used as a first step in purification. Fraction 15 (purple bar) contained the two isobaric conformers PcaTX-1a and PcaTX-1b. (**B**) Reverse-phase chromatography was used for further toxin purification. PcaTX-1a can be seen in Fraction 33 (red bar) and PcaTX-1b can be seen in Fraction 35 (blue bar). Figure S2 is provided to show the result of these purifications.

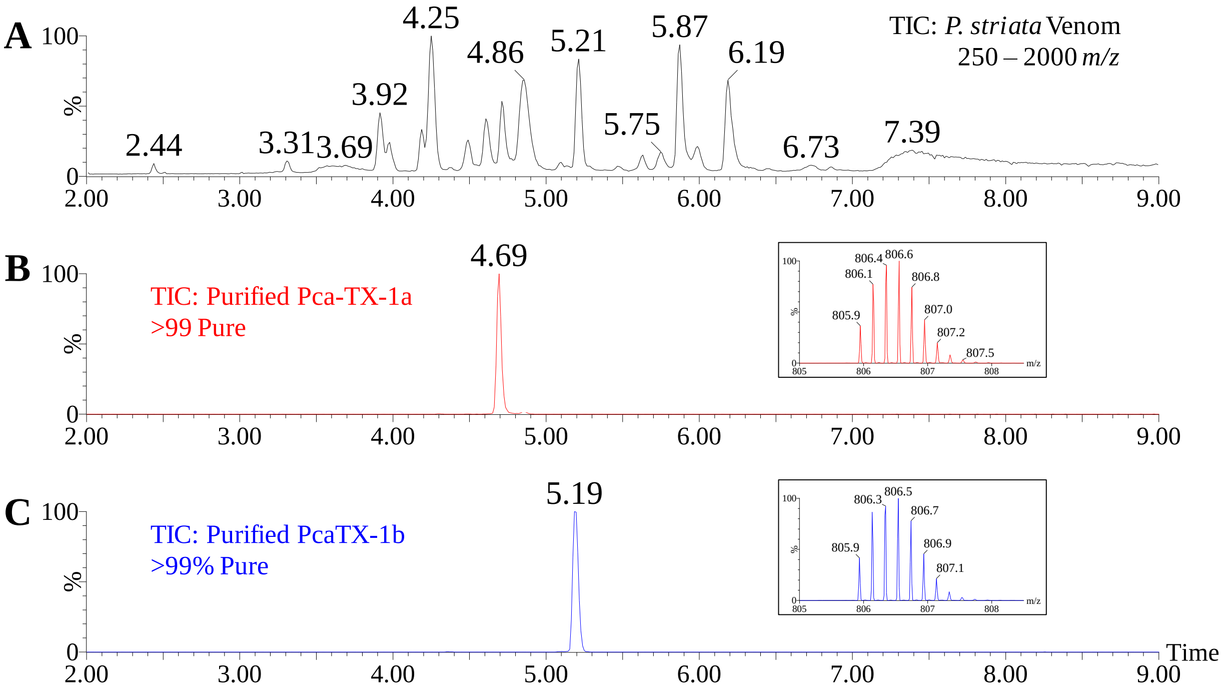

Figure S2. Representative purified peptides. (**A**) The TIC for *Poecilotheria striata* venom with a range from 250–2000 *m*/*z* is provided to show the overall complex and diverse mixture of molecules in the venom primarily made up of small peptides ranging from 3–6 kDa. Fractions of whole venom that were chromatographically separated by size exclusion chromatography were rechromatographed by reverse-phase chromatography to provide pure native analogs for physiological assays. Representative TIC for (**B**) PcaTX-1a and (**C**) PcaTX-1b are provided to show the peptides were purified to greater than 99%. The inset shows the $[M + 5H]^{5+}$ molecular ion cluster to verify the compound's identity. The red color used indicates the presence of *isoAsp* and the blue color used indicates the presence of *Asp*.
